# The Pathogenesis and Diagnosis of *Pneumocystis jiroveci* Pneumonia

**DOI:** 10.3390/jof8111167

**Published:** 2022-11-05

**Authors:** Anna Apostolopoulou, Jay A. Fishman

**Affiliations:** 1 Division of Infectious Diseases, Massachusetts General Hospital, Harvard Medical School, Boston, MA 02114, USA; 2 MGH Transplant Center, Massachusetts General Hospital, Harvard Medical School, Boston, MA 02114, USA

**Keywords:** *Pneumocystis jiroveci*, *Pneumocystis pneumonia*, fungal infection, cytomegalovirus, HIV, AIDS, corticosteroids, organ transplantation, hematopoietic stem cell transplantation

## Abstract

*Pneumocystis jiroveci* remains an important fungal pathogen in immunocompromised hosts. The environmental reservoir remains unknown. Pneumonia (PJP) results from airborne transmission, including in nosocomial clusters, or with reactivation after an inadequately treated infection. *Pneumocystis pneumonia* most often occurs within 6 months of organ transplantation, with intensified or prolonged immunosuppression, notably with corticosteroids and following cytomegalovirus (CMV) infections. Infection may be recognized during recovery from neutropenia and lymphopenia. Invasive procedures may be required for early diagnosis and therapy. Despite being a well-established entity, aspects of the pathogenesis of PJP remain poorly understood. The goal of this review is to summarize the data on the pathogenesis of PJP, review the strengths and weaknesses of the pertinent diagnostic modalities, and discuss areas for future research.

## 1. Introduction

*Pneumocystis* is an ascomycetous fungus, formerly classified as a protozoan but re-classified based on ribosomal RNA analysis that showed greater homology with the fungi [1,2]. The natural reservoir of infection remains unknown. *Pneumocystis carinii*, renamed *P. jiroveci* (or *jirovecii*), was first described as a human pathogen in 1942 by van der Meer and Brug, who described three fatal cases of interstitial pneumonia in two infants and one young adult [3]. This was followed by a report of interstitial plasma cell pneumonia in malnourished infants in 1952 by Vanek and Jirovec [4]. Several case reports of the infection in premature infants were reported in the pediatric literature of the 1950s. By the 1970s, *Pneumocystis* pneumonia was reported in patients with congenital immunodeficiencies, hematologic malignancies, hypogammaglobulinemia and in patients receiving corticosteroids or cytotoxic agents for the treatment of lymphoproliferative disorders [5,6,7,8]. Many cases were described with childhood malnutrition in the wake of war. In 1981, PJP (abbreviated as PCP or *Pneumocystis carinii* pneumonia until that time) became the first AIDS-defining opportunistic infection by the U.S. Centers for Disease Control and Prevention (CDC) [9]. In the early days of solid organ transplantation, PJP was diagnosed post-mortem in solid organ recipients at a time when high doses of prednisone were employed for immunosuppression [10]. Although *Pneumocystis jiroveci* pneumonia (PJP) is common in immunocompromised hosts, the pathogenesis of infection remains incompletely understood. This review will discuss host factors that predispose to PJP, review available diagnostic methods and propose areas for future investigation.

## 2. Phylogeny

Controversy has existed with the phylogenetic assignment of *P. jiroveci* to the taxonomic kingdom of fungi. This occurred based on ribosomal RNA and mRNA sequences, including the separate genes encoding the thymidylate synthase and dihydrofolate reductase of *P. jiroveci* and β-glucan in the cyst cell wall. Such observations should have implications for disease transmission and therapy. The environmental reservoir remains unknown. The neutral lipid fraction of *P. jiroveci* includes a variety of phytosterols shared by plants and fungi, including the plasmodial slime molds such as *Physarum* species [11]. The plasmodial slime molds form a diverse group of heterotrophic, mobile and largely unicellular organisms that are abundant in nature. Many such organisms, including Pneumocystis, develop genetic rearrangements as occur for the species-specific major surface glycoprotein genes (gp120, gpA, MSG) [12,13,14]. It is unclear whether MSG shifts in *Pneumocystis* are driven by the host immune response or by additional factors, such as the availability of nutritional resources from the host cells to which they bind [15,16,17,18,19,20]. The cell wall contains glucose, mannose, cholesterol and phytosterols but no ergosterol, accounting for the absence of susceptibility to azole and polyene antifungal antibiotics [11,21]. In *Pneumocystis*, the absence of protein mannosylation found in other fungi may help avoid uptake via mannose-binding receptors [22,23].

## 3. Pathogenesis

### 3.1. Pneumocystis Life Cycle and Transmission of Infection

Little is known about the natural reservoir of *Pneumocystis jiroveci.* In mammalian lungs, *Pneumocystis* exists in three morphologically distinct forms: trophic forms (or trophozoites), sporozoites and mature cysts. The infectious process begins with the attachment of the trophozoites to the alveolar type I pneumocytes. Trophozoites are eukaryotic cells with cytoplasmic projections (filopodia), which mediate attachment to alveolar type I pneumocytes. Trophozoites represent the predominant form of the organism in in vivo and in vitro culture systems. In the lungs, *Pneumocystis* has a biphasic life cycle featuring (a) an asexual phase, which consists of the binary fission of trophozoites, and (b) a sexual phase, during which trophozoites fuse, leading to the formation of cysts. Haploid trophozoites conjugate into diploid early sporozoites, which undergo meiotic division followed by mitotic replication. This results in the formation of late sporozoites containing eight nuclei (or spores). The maturation of late sporozoites leads to the evolution of thick-walled cysts [14,24]. β-(1,3)-D-glucan synthetase generates cyst walls rich with β-(1,3)-D-glucan, which is a target of binding, activation, and phagocytosis by macrophages. β-1,3-glucan is absent in trophozoites [22,23].

In rodent models of PJP, treatment with anidulafungin (an echinocandin that inhibits β-(1,3)-D-glucan synthase) selectively depleted cysts but not trophozoites. Anidulafungin-treated mice were unable to transmit infection, suggesting that cysts may be the agents of disease transmission [25]. In inoculated murine models using *Pneumocystis murina* [26], prophylaxis with the echinocandin rezafungin reduced both trophozoites and cysts (called asci) in infected mice and was effective in preventing infection in steroid-treated mice [27,28].

Animal data support transmission via the airborne route [29,30]. This has been demonstrated in both immunocompetent and immunocompromised murine models when *Pneumocystis*-infected and uninfected mice are housed together [31,32,33]. The observation of *Pneumocystis* replication in the lungs of immunocompetent animals without causing disease has led to the hypothesis that immunocompetent hosts can act as a reservoir for the infection [33]. In humans, nosocomial outbreaks of PJP have been reported among solid organ transplant recipients; in several studies, genetic sequencing has demonstrated human-to-human transmission, including from asymptomatic carriers [34,35,36,37,38,39]. Data are inconclusive regarding the possible role of healthcare workers in the nosocomial spread of PJP [40,41].

### 3.2. Primary Infection versus Reactivation of Latent Infection

In an early study of immunocompetent children, two-thirds had positive IgG against *Pneumocystis* by age four, suggesting primary infection in early childhood [42]. Similarly, in a 2-year prospective cohort of healthy newborns, *Pneumocystis jiroveci* DNA was isolated from nasopharyngeal samples obtained upon clinical suspicion of upper or lower respiratory tract infection in the study population. Further, 84.8% of infants had seroconverted at the conclusion of the study [43]. Such observations led to the hypothesis that PJP is a result of the reactivation of latent infection from childhood. On the other hand, genetic typing studies of the *Pneumocystis jiroveci* ribosomal RNA in patients with recurrent PJP PNA showed both reinfection and reactivation of a previously acquired infection can occur [44,45]. In steroid-treated rats, the infection does not recur despite immunosuppression in animals maintained in air-filtration cages following adequate treatment of infection, suggesting that reinfection is an important mechanism of disease (J. Fishman and M. Bartlett, unpublished data).

### 3.3. Host Risk Factors

Clinically significant PJP is observed only in hosts with congenital or acquired immune deficits. T-lymphocyte deficiencies, as well as defects in macrophage function, are implicated in the pathogenesis of PJP. Congenital, acquired, or idiopathic CD4+ T cell lymphopenia is a known risk factor for PJP [46,47,48,49,50,51]. In patients with HIV, the evolution of highly effective antiretroviral therapy (ART) has led to a decline in the incidence of PJP [52,53]. Prophylaxis against *Pneumocystis* is generally recommended in patients with a CD4+ cell count of <200 cells/mL [54]. Nevertheless, recent data from the Collaboration of Observational HIV Epidemiological Research Europe (COHERE) cohort have demonstrated that both primary and secondary PJP prophylaxis can be safely withdrawn (or withheld) in virologically suppressed patients on ART and CD4+ counts of 100–200 cells/mL as the incidence of PJP in this population is low [55,56].

In non-HIV patients, the most common risk factor for PJP is steroid therapy [57]. The correlation between steroids and PJP was described as early as the 1960s in a case series of children who developed PJP 50–90 days after the onset of steroid treatment for lymphoproliferative disease [6]. The author of the series noted that clinically apparent infection manifested when the steroid dose was reduced or steroid treatment was completed, suggesting clinical signs due to re-emerging immune responses; conversely, resumption of prior steroids led to clinical improvement in some subjects. In animal models, severe PJP was reproduced in rats inoculated with *Pneumocystis jiroveci* following systemic steroids [58]. The risk of PJP in steroid-treated patients is dose-dependent; high doses (>30 mg/day of prednisone) or lower doses (15–30 mg/day) for more prolonged periods (≥4 weeks) have been associated with PJP [59,60].

In patients with lymphoproliferative diseases, humoral and cellular immune dysfunction related to the underlying disease, as well as immunosuppression related to treatment, are major predisposing factors for PJP. Absolute lymphopenia and prolonged neutropenia are known risk factors for PJP [61]. Among hematological malignancies, patients with acute lymphoblastic leukemia (ALL) and patients who have undergone allogeneic stem cell transplantation are among the highest risk groups for PJP [62,63]. In the latter group, established risk factors include immunosuppressive treatment for graft-versus-host disease (GVHD). Guidelines recommend extending PJP prophylaxis beyond the first 6 months post-transplantation with GVHD therapy [64]. Immunosuppressive medications such as purine and pyrimidine analogues (fludarabine, cytarabine) are associated with lasting T cell lymphopenia and increased risk for PJP. PJP prophylaxis is recommended for patients who are receiving these agents [62,65]. Lymphocyte-depleting agents, including the anti-CD20 monoclonal antibodies (e.g., rituximab, obinutuzumab, ofatumumab) and the combined T and B cell-depleting anti-CD52 alemtuzumab have been associated with PJP [66,67,68,69,70]. Anti-CD20 monoclonal antibodies are associated with protracted B cell depletion through complement-mediated and cell-mediated cytotoxicity, lasting at least 6 to 9 months after each infusion [71]. Rituximab is also associated with late-onset neutropenia [72,73]. In a small retrospective case-control study including HIV-negative patients hospitalized with PJP, PJP was found to be independently associated with the use of rituximab, suggesting a role for antibodies in protection against PJP [67]. European guidelines recommend PJP prophylaxis in patients receiving alemtuzumab and rituximab either alone or in combination with other immunosuppressants for cancer-directed treatment (e.g., R-CHOP) [64,74]. Prolonged B cell depletion due to CD19-targeted chimeric antigen receptor-modified T cell (CAR-T) immunotherapies has also been associated with some cases of PJP [75,76]; prophylaxis may limit the incidence of PJP in this group. Among newer agents, the phosphoinositide 3-kinase inhibitors (e.g., idelalisib, duvelisib, copanlisib) may be associated with a higher risk of PJP [77], especially in combination with bendamustine and rituximab [78]. Venetoclax, which promotes apoptosis in malignant cells via inhibition of the B cell lymphoma 2 (BCL-2) protein, causes significant neutropenia and has been associated with fungal infections, including PJP [79]. On the other hand, ibrutinib, which inhibits Bruton’s tyrosine kinase and thus B cell proliferation, is less frequently associated with PJP [80,81,82].

In the solid organ transplant population, the risk of PJP relates to the intensity of the immunosuppression. Without PJP prophylaxis, this risk is highest in the first six months post-transplant, during periods of augmented immunosuppression (e.g., high-dose steroids or anti-thymocyte globulin for management of allograft rejection) and up to 3–6 months afterwards. The routine use of PJP prophylaxis in the early post-transplant period has dramatically decreased the incidence of PJP in the first six months to a year after transplantation. The risk seems to increase in the second-year post-transplantation, especially in the setting of allograft rejection, CMV infection, age ≥ 65 years and lymphopenia; the association between CMV infection and PJP has been found to be statistically significant in several studies [37,83,84,85,86,87,88]. Further, in a large retrospective cohort study of *Pneumocystis jiroveci* and respiratory co-infections, only CMV co-infection was independently associated with 90-day mortality [89]. With regards to other risk factors, in a recently published case-control study, the co-stimulatory blocker belatacept was associated with an increased risk of PJP, though it is unclear whether this association is independent of other risk factors, including viral co-infections [90]. The syndrome of “rapamycin lung” is a diffuse pneumonitis observed with mTOR (mammalian target of rapamycin) inhibitors (e.g., everolimus, sirolimus and temsirolimus), generally in concert with other infections, including community-acquired respiratory viruses and *Pneumocystis*. Resolution requires cessation of the mTOR agent and treatment of infection.

PJP has also been described in association with the anti-tumor necrosis factor-α (TNFα) monoclonal antibody infliximab, which is broadly used in patients with autoimmune disease and inflammatory bowel disease [91,92,93,94]. In the largest available series [91], PJP occurred at a mean of 21 days after the administration of the drug and was associated with significant mortality (27%).

## 4. Diagnosis

### 4.1. Clinical Presentation

PJP should be considered in the differential diagnosis of any immunocompromised individual with fever, dyspnea with hypoxemia beyond the radiographic appearance, and a nonproductive cough. Recent changes in immunosuppression or viral co-infections are common, as are comorbid conditions such as pulmonary edema or lung allograft dysfunction. Progression is acute to subacute in non-AIDS patients, while the initial episode of PJP in AIDS often has a more gradual evolution (often 2 to 5 weeks) with prominent constitutional symptoms. Extrapulmonary pneumocystosis is uncommon and is seen most often in untreated AIDS.

### 4.2. Radiography

**Figure 1:**
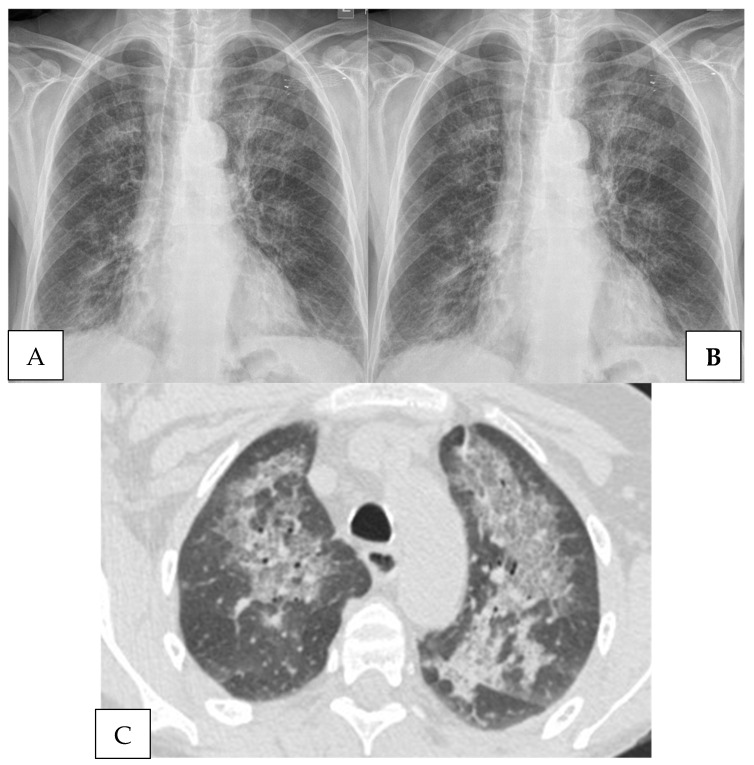
A 65-year-old woman 18 months after deceased donor kidney transplantation presents to emergency room with increasing dyspnea over the past three weeks and new fevers. She completed trimethoprim-sulfamethoxazole prophylaxis one year prior to admission. She is hypoxic with decreased breath sounds bilaterally and fine diffuse crackles, LDH is 416, β-1,3-glucan assay positive (>500), and induced sputum sample positive of *Pneumocystis jiroveci* by immunofluorescence. (**A**). Baseline chest radiograph. (**B**). Admission chest radiograph with slightly increased upper lobe ground glass markings. (**C**). Chest CT scan without intravenous contrast reveals a mixed pattern of patchy ground-glass opacities and scattered dense pulmonary infiltrates with associated septal thickening.

The radiographic appearance of PJP is classically a bilateral interstitial pneumonia with diffuse patchy consolidative and ground-glass opacities (Figure 1). PJP often presents with atypical features with cavitary or nodular features; atypical features are amplified with co-infections such as CMV or adenovirus. In general, no radiographic pattern is pathognomonic for PJP, and radiographic mimics exist. Those include but are not limited to drug-induced pneumonitis associated with mTOR inhibitors, immune checkpoint inhibitor therapy-related pneumonitis [95], severe viral pneumonia, radiation pneumonitis, and eosinophilic pneumonia associated with chimeric antigen receptor T cell (CAR T) therapy [96].

### 4.3. Microbiology

**Figure 2:**
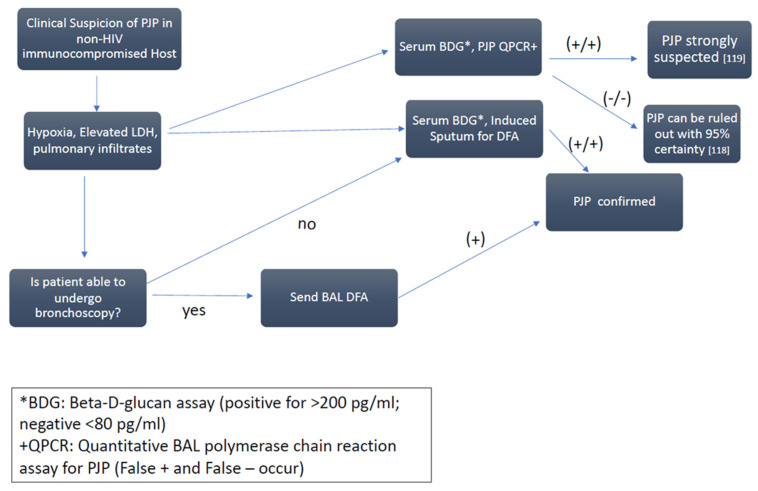
Approach to the diagnosis of Pneumocystis pneumonia. In immunocompromised patients at risk for *Pneumocystis jirovecii* pneumonia (PJP), early antimicrobial therapy is initiated based on clinical signs and symptoms, radiology, and available laboratory data [118,119]. Microbiological demonstration of organisms on bronchoalveolar lavage (BAL) or induced sputum specimens should not delay treatment. Other processes, including co-infections, may alter the radiologic picture or mimic PJP clinically.

*Pneumocystis* is grown in vitro on a feeder cell layer of pulmonary epithelial cells [97]; this system is not useful for routine microbiological diagnosis. Traditionally, diagnosis has relied on direct microscopic visualization of *Pneumocystis* in induced sputum, bronchoalveolar lavage specimens, and transbronchial or open lung biopsy specimens (Figure 2). Both conventional polychrome stains and immunofluorescent staining via monoclonal antibodies to *Pneumocystis*, also known as direct immunofluorescent antibodies (DFA), are employed. Conventional stains include Gomori methenamine silver (GMS), toluidine-blue O, calcofluor white and Gram–Weigert, which stain the cell wall of the cysts. Other available stains (Giemsa, Diff-Quik, and Wright) detect the sporozoites and trophozoites but do not stain the cyst wall. DFA performed on induced sputum or bronchoalveolar lavage is the diagnostic modality of choice; conventional stains are seldom used in resource-rich settings. Immunofluorescent antibody stains require less technical skill for test interpretation, stain both cysts and trophozoites and are more reproducible than conventional stains [98]. Older data suggested that conventional stains were adequate with the heavier organism burdens found in patients with HIV/AIDS compared to other immunocompromised patients [99]. In a multicenter study assessing the performance of three conventional staining methods (calcofluor white, Diff-Quik, GMS) vs. DFA for the detection of *Pneumocystis* in 313 respiratory specimens (mostly BAL), the DFA was most sensitive (90.8% as opposed to 50–80% for the conventional stains) but less specific (94.7% as opposed to >99% for the conventional stains) [100].

PCR-based amplification assays for the detection of *Pneumocystis* were introduced in the 1990s, allowing for higher sensitivity when compared to DFA [101,102,103]. Data suggest that the superior sensitivity of PCR over DFA and conventional stains is notable with the lower organism load of induced sputum or oral wash specimens rather than BAL [102,104,105]. However, PCR cannot differentiate between infection and colonization, leading to higher rates of false positive assays; further, assay sensitivity depends on gene target selection [103,106]. In order to improve the specificity and positive predictive value of molecular assays, real-time quantitative *Pneumocystis* PCR (qPCR) assays were developed, with the rationale that the detection of a higher organism burden would be associated with true infection as opposed to colonization [107]. Some studies have proposed cut-off values of >1450 or >1900 pathogens/mL for the diagnosis of PJP, with positive predictive values of 98% and 100%, respectively [108,109]. However, these cut-offs are not standardized, and prospective studies are needed to validate such results. Some experts recommend using *P. jirovecii* qPCR as an adjunct in conjunction with microscopy on BAL to increase the diagnostic yield [110,111].

In several small retrospective cohorts, next-generation sequencing enables the diagnosis of co-infections from the same specimen (e.g., CMV and PJP) [112,113,114,115,116]. A recent study investigated the diagnostic accuracy of the non-invasive blood cell-free DNA (cfDNA) PCR assay in a large cohort of immunocompromised patients. Using the DFA as the gold standard (“proven PJP”), the authors found that the *Pneumocystis jiroveci* cfDNA test was 100% sensitive and 93.4% specific in a small cohort with proven disease [117]. However, performance was less robust in patients with lower organism burdens. This modality may provide adjunctive information in patients unable to tolerate sputum induction, bronchoscopy or lung biopsy.

### 4.4. Other Laboratory Evaluation

Non-specific tests useful as adjuncts include the enzyme lactate dehydrogenase (LDH) and the serum (1,3)-β-D-glucan assay (BDG). Serum LDH is usually elevated (>300 IU/mL) in patients with PJP reflecting diffuse pneumonia (Figure 2). BDG is a non-specific fungal marker that has high sensitivity but low specificity for PJP and may be used as a surrogate (rather than a stand-alone) test for a suspected diagnosis of PJP. In a systematic review and meta-analysis of 22 studies including both patients with and without HIV, the pooled overall sensitivity of BDG was 91% (94% in patients with HIV vs. 86% in patients without HIV). A negative predictive value of BDG at <80 pg/mL (the manufacturer’s recommended cut-off) was 95% when the pre-test probability was intermediate (50%) [118]. In a large study of patients with cancer and a clinical syndrome consistent with PJP for patients with a positive BAL PJP PCR, a BDG > 200 pg/mL had 100% specificity and 100% positive predictive value for PJP [119].

### 4.5. Histopathology

The classic histologic appearance of PJP involves foamy eosinophilic exudates within the alveoli. Rarely, *Pneumocystis* can trigger a granulomatous inflammatory response and the formation of necrotizing (or less commonly non-necrotizing) granulomas. Histologic findings of “granulomatous PJP” include *Pneumocystis* organisms found within the granulomas, epithelioid histiocytes and a surrounding rim of lymphocytes [120]. Most organisms are trophozoites (not seen with fungal silver stains). Macrophage dysfunction due to CMV co-infection or GCSF deficiency (in mice) increases cyst counts.

## 5. Conclusions and Future Directions

Several aspects of the pathogenesis of PJP remain poorly understood. These include the unknown natural reservoir of the fungus and the potential role of immunocompetent hosts as reservoirs or intermediate hosts of infection. With studies reporting nosocomial outbreaks of PJP amongst immunocompromised hosts, optimal monitoring and prevention strategies merit consideration. The landscape of primary and secondary PJP prophylaxis is changing. The COHERE study demonstrated the efficacy of virologic suppression for primary and secondary PJP prevention in patients with advanced, treated HIV [56]. In immunocompromised patients without HIV, tools are required for the individualization of prophylaxis based on an assessment of the net state of immunosuppression; such assays are under development. Newer immunosuppressive agents, including new biologics, cellular therapies, and oncology drugs, merit study. In solid organ transplant recipients, most transplant centers have institutional policies regarding routine PJP prophylaxis to prevent early onset PJP. However, further research is required to better understand the optimal deployment of prophylaxis for late-onset PJP during periods of increased susceptibility (e.g., after allograft rejection or CMV infection). The pharmacotherapy of PJP has not changed substantially in the past few decades; a phase III clinical trial (ReSPECT) is investigating rezafungin for the prevention of PJP in patients undergoing allogeneic bone marrow transplantation. An international consensus could be developed for standardized diagnostic criteria and prophylactic strategies for PJP as for other invasive fungal infections. The diagnosis of PJP requires a high index of suspicion in immunocompromised patients with compatible clinical syndromes and laboratory and radiographic findings.

## Data Availability

Not applicable.
